# Cervical radiculopathy: Study protocol of a randomised clinical trial evaluating the effect of mobilisations and exercises targeting the opening of intervertebral foramen [NCT01500044]

**DOI:** 10.1186/1471-2474-13-10

**Published:** 2012-01-31

**Authors:** Pierre Langevin, Jean-Sébastien Roy, François Desmeules

**Affiliations:** 1 Physio interactive Clinics, 3520, rue de l'Hêtrière, Saint-Augustin-de-Desmaures, (QC), Canada, G3A 0B4; 2 Department of Rehabilitation, Faculty of Medicine, Laval University, Pavillon Ferdinand-Vandry, 1050, avenue de la Médecine, Quebec City (QC), Canada, G1R 1P5; 3 Centre for Interdisciplinary Research in Rehabilitation and Social Integration, Quebec Rehabilitation Institute, 525, Boulevard Wilfrid Hamel, Quebec City (QC), Canada, G1M 2S8; 4 School of Rehabilitation, University of Montreal, CP 6128 Succursale Centre-Ville, Montreal (QC), Canada, H3C 3J7; 5 Orthopaedic Clinical Research Unit, Maisonneuve-Rosemont Hospital Research Center, 5415, Boulevard l'Assomption, Montreal (QC), Canada, H1T 2M4

**Keywords:** Cervical, Radiculopathy, Neck pain, Exercises, Mobilisations, Intervertebral foramen

## Abstract

**Background:**

Cervical radiculopathy is a common form of neck pain and has been shown to lead to severe disability. Clinical rehabilitation approaches for cervical radiculopathies commonly include exercise and manual therapy interventions targeting the opening of intervertebral foramen, but evidence regarding their effectiveness is scarce. The primary objective of this randomised clinical trial is to compare, in terms of pain and disability, a rehabilitation program targeting the opening of intervertebral foramen to a conventional rehabilitation program, for patients presenting acute or subacute cervical radiculopathies. The hypothesis is that the rehabilitation program targeting the opening of intervertebral foramen will be significantly more effective in reducing pain and disability than the conventional rehabilitation program.

**Methods/Design:**

This study is a double-blind (participants and evaluators blinded) randomised clinical trial that will allow the comparison of patients with a cervical radiculopathy randomly assigned to one of two groups: one group will receive a 4-week rehabilitation program targeting the opening of intervertebral foramen, and the second group will receive a 4-week conventional rehabilitation program. Thirty-six subjects with cervical radiculopathy will be recruited from participating medical and physiotherapy clinics and will be evaluated at baseline, at the end of the 4-week program and four weeks following the end of the program. The primary outcome measure will be the validated Neck Disability Index questionnaire. Secondary outcome measures will include the short version of the Disabilities of the Arm, Shoulder and Hand questionnaire, a numerical pain rating scale, cervicothoracic mobility and patients' perceived global rating of change. During the 4-week rehabilitation program, each participant will take part in eight physiotherapy treatment sessions (2 session/week) and will perform a home exercise program. A mixed-model, 2-way ANOVA will be used to analyze the effects of the rehabilitation programs.

**Discussion:**

Control trials are needed to define ideal intervention approaches in rehabilitation for this population. This randomised clinical trial will be the first study that directly compares a rehabilitation program targeting the opening of intervertebral foramen to a conventional rehabilitation program for patients with cervical radiculopathy. The results of this study may help to establish best clinical practice guidelines for this patient population.

**Trial Registration:**

ClinicalTrials.gov: NCT01500044

## Background

Cervical or neck pain is a general term used to designate any musculoskeletal disorder in the cervical region. Various pathologies encompass that generic definition and are most commonly related to degenerative changes or inflammation of cervical structures such as intervertebral discs, articular facets joints or nerve roots. Neck pain is a very common, disabling and costly condition [1-3]. According to a review by the Neck Pain Task Force pertaining the prevalence of neck pain in industrialised countries, annual prevalence is situated within 30 to 50% in adult populations [1]. In accordance with these results, in Canada, a bi-annual prevalence of 54% has been reported [1].

Cervical radiculopathy forms an important subgroup of neck disorders and, although less prevalent than general neck pain, it has been shown to lead to more severe pain and disability [4-9]. Cervical radiculopathy primarily results from an inflammation of a cervical nerve root induced by a lesion reducing the intervertebral foramen [10]. This reduction is primarily induced by a herniated disc or a degenerative lesion of zygapophysial joints [11-13]. Typical symptoms of cervical radiculopathy include pain in the cervical or periscapular region and in the upper limb, as well as neurological signs such as paresthesia, numbness, weakness and loss of reflexes in the affected nerve root distribution [14].

The diagnosis of cervical radiculopathy is commonly made through patient history and physical examination. Wainner et al. have shown that a positive response to the following four clinical tests results in a high predictive value for a diagnosis of cervical radiculopathy [15]: 1- cervical distraction test, 2- upper limb tension test (ULTT), 3- Spurling test, and 4- ipsilateral cervical rotation reduced by more than 60°. If all four of these tests are positive, the positive likelihood ratio (LR+) of having a cervical radiculopathy is 30. If three out of these four tests are positive, the LR+ decreases to 6. According to Straus et al., a LR+ superior to 10 is considered large, and between 5 and 10 moderate; thus, it increases the possibility that the impairment in question is present [16]. Hence, by combining these clinical tests, the possibility of obtaining a good clinical diagnostic accuracy in patients presenting signs and symptoms compatible with cervical radiculopathy is high.

While the clinical diagnostic process of cervical radiculopathies is relatively well documented, studies evaluating the effectiveness of rehabilitation interventions remain sparse [17-20]. Published in 2010, a systematic review by Miller et al., pertaining to the effects of manual therapy and exercises on the treatment of neck pain, concluded that there is little evidence supporting the efficacy of these modalities in the treatment of cervical radiculopathies [17]. Of the 17 randomised clinical trials (RCT) included in this systematic review, only three included subjects who presented radicular signs. Furthermore, in these three RCTs, subjects with or without radicular signs were combined for the statistical analysis used to evaluate the effects of the intervention. The authors of the systematic review concluded that, for neck pain, a combination of cervicothoracic mobilisations and exercises is the most effective rehabilitation approach to reduce pain and disability [17]. No specific recommendation was, however, brought forth for cervical radiculopathies. Three RCTs published in 2009, but not included in the systematic review by Miller et al., also evaluated the effects of rehabilitation approaches for the treatment of cervical radiculopathy [21-23]. Kuijper et al. randomised 205 patients suffering from cervical radiculopathy within 3 groups: a "*cervical collar*" approach, an "*active physiotherapy*" approach, and a "*wait and see*" approach [21]. The "*active physiotherapy*" approach involved mobilisations and stabilisation exercises; whereas the "*cervical collar*" approach included the use of a semi-hard cervical collar worn at all times for three weeks, then gradually weaned for three additional weeks. Three and six weeks into the intervention, a diminution of arm and neck pain was observed in the "*cervical collar*" and "*active physiotherapy*" groups. Functional improvement was also observed in both experimental groups at three weeks, but was only noted in the "*cervical collar*" group at six weeks. While this study's conclusions seem to favour the "*cervical collar*" approach, it remains a controversial treatment option. According to the Quebec Task Force, cervical collar should be avoided due to its passive and decondition properties, and because it has been shown to hinder neck pain recuperation following motor vehicle accidents [24]. These initial recommendations regarding the potential drawbacks of cervical collar use have recently been generalised to encompass all types of neck pain [19,25]. Finally, two other studies have evaluated the effect of intermittent tractions on patients suffering from cervical radiculopathy [22,23]. They have, however, obtained contradictory results: one demonstrated that the addition of tractions to a conventional intervention does not increase treatment efficacy [22], whereas the other claimed that tractions supplementing a conventional intervention improves cervical and radicular pain, in comparison to a conventional intervention [23].

Numerous other approaches are commonly utilised in clinical settings, but a formal demonstration of their efficacy remains to be shown. Clinical approaches for cervical radiculopathies commonly include interventions targeting the opening of intervertebral foramen [26]. It is well recognised that cervical movements causing the opening of intervertebral foramen, such as flexion, rotation and lateral flexion contralateral to the nerve root, increase the volume of the foramen, and consequently might decompress a swollen nerve root [27-31]. Inversely, movements of extension, rotation and lateral flexion ipsilateral to the nerve root close the intervertebral foramen around the root. Thus, for acute and sub-acute radiculopathies, intervention programs should include treatment modalities that allow the opening of the intervertebral foramen. On the other hand, movements and positions that lead to intervertebral foramen closure should be avoided. However, no studies have evaluated the effects of a treatment approach that specifically take into consideration these biomechanical principles. Due to the important incapacities related to cervical radiculopathy, and to the few studies pertaining to the efficacy of rehabilitation in this population, we believe in the importance of better understanding the potential of cervical mobilisations and exercises that lead to the opening of the intervertebral foramen. The primary objective of this RCT is to compare, in terms of pain and disability, a rehabilitation program targeting the opening of intervertebral foramen to a conventional rehabilitation program, in patients presenting acute or subacute cervical radiculopathies. Based on biomechanical principles, our hypothesis is that the rehabilitation program targeting the opening of intervertebral foramen will be significantly more effective in reducing pain and disability than the conventional rehabilitation program, mainly at the 4-week evaluation. In considering the passage of time, the differences between the two interventions should be less important at 8-week.

## Methods/Design

### Study Design

This double-blind randomised clinical trial will allow the comparison, in terms of pain and disability, of patients presenting a cervical radiculopathy which will have been randomly assigned to one of the two intervention groups: the first group (n = 18) will receive a 4-week rehabilitation program targeting the opening of intervertebral foramen, the second group (n = 18) will receive a 4-week conventional rehabilitation program. Participants will be evaluated on three separate occasions: at baseline (week 0), at the end of the 4-week program (week 4), and four weeks following the end of the program (week 8).

### Participants

Thirty-six subjects with cervical radiculopathy will be recruited from participating medical and physiotherapy clinics in the Quebec City (Canada) area. The study will include patients who meet the following inclusion criteria: 1- age greater than 18 years and less than 65 years; 2- pain, paresthesia or numbness in the upper-limb with cervical or periscapular pain for less than 3 months; 3- at least one neurological sign (dermatomes, myotomes or reflexes) of an inferior motoneuron lesion to the upper-limb; and 4- positive responses to at least 3 of the 4 following clinical tests: Spurling Test, Upper Limb Tension Test, Cervical Distraction Test, and less than 60° of cervical rotation on the impaired side. The following exclusion criteria will be used for this study: 1- prior surgery to the cervicothoracic spine; 2- bilateral upper-limb symptoms; 3- signs of superior motoneuron impairments (bilateral paresthesia, hyperreflexia, spasticity); 4- cervical spine injection in the previous four weeks; 5- current use of steroidal anti-inflammatory drugs; and 6- financial compensation for the cervical condition. Before the beginning of the study, physiotherapists and physicians from participating clinics will be contacted to explain the goals of the study and eligibility criteria. Patients presenting pain, paresthesia or numbness of the upper limb with or without cervical pain will be identified from the participating clinics. The treating physiotherapist or physician will explain the goal of the study to the identified patients to enquire about their interest in participating to the study. Those interested will be contacted by phone by a member of the research team for a preliminary screening of their condition (sociodemographic, symptomatology, medication criteria). If they meet the eligibility criteria, and after providing written informed consent, they will be evaluated by an independent physiotherapist, at the Center for Interdisciplinary Research in Rehabilitation and Social Integration (CIRRIS), who will perform physical tests to determine their full eligibility. Demographic data (gender and age) of patients who refuse to participate and of ineligible patients willing to participate will be collected to evaluate potential selection bias. The Institutional Review Board at Quebec Rehabilitation Institute has granted approval for the study.

### Examination procedure and randomisation

After recruitment, the eligible participants will take part in the baseline evaluation. They will complete a questionnaire on sociodemographic, symptomatology and comorbidity, and three self-reported questionnaires on pain and disability: the Neck Disability Index (NDI), the short version of the Disabilities of the Arm, Shoulder and Hand Questionnaire (*Quick*DASH), and a numerical pain rating scale (NPRS). Finally, they will undergo a physical examination of the cervicothoracic spine, which will include the evaluation of cervicothoracic range of motion using a CROM (cervical inclinometers).

After baseline evaluations, assignation of patients to either the conventional or experimental intervention will be performed. To reduce contamination bias, the two programs will be given in different clinics. An independent research assistant will open the randomisation envelope indicating the participant's assignment to the group of clinics that corresponds to the experimental or control group. The research assistant will be blind to the baseline evaluation results and to the assignation of which clinic is providing which intervention. The research assistant will give the list of the clinics providing the rehabilitation program assigned to the participant, and the participant will choose the clinic of their choice. Random number generator will be used to establish randomisation lists prior to the initiation of the study. A member of the research team not involved with data collection will generate the randomisation list. To make sure that two equal groups of 18 subjects will be obtained, blocked randomisation will be used (block size of 6). Three physical therapy clinics will provide the control rehabilitation program and three different clinics the experimental rehabilitation program.

At the end of the 4-week rehabilitation program (maximum 3 days after the last treatment session), and four weeks after the end of the program, the three self-reported questionnaires (NDI, *Quick*DASH, and NPRS), as well as cervicothoracic range of motion will be revaluated. Furthermore, at the two follow-up evaluation sessions, a question pertaining to global change since the initial session (*Global rating of Change *[GRC]) will be completed. An independent physiotherapist, blind to the group assignment, will perform the evaluations according to standardised procedures.

### Blinding

A double-blind design will be used, in which the participants and evaluators will be blinded. Due to the nature of the intervention, it is not possible to blind the treatment provider to the treatment given. However, we think that the participants can be blinded since they will not be aware of the precise type of mobilisations received and to reduce potential contamination bias, the two programs will be given in different clinics. To evaluate the effectiveness of the blinding, the participants and the evaluator will complete a questionnaire related to their opinion of the allocation at the week-4 follow-up evaluation. We will blind the evaluator performing the outcome evaluations and the evaluation session will take place outside the treating clinics. The evaluator and treatment providers will be different physiotherapists. Patients will be instructed not to discuss the specific mobilisation techniques and exercises received with the evaluator. At the end of study, participants will be informed of their group allocation.

### Outcome measures (dependent variables)

The NDI questionnaire has been chosen as the primary outcome measure. All evaluations will be undertaken at the CIRRIS. Here is a description of the outcome measures:

*Neck Disability Index: *The NDI is a 10-item questionnaire that measures a patient's self-reported neck pain related disability. Questions include activities of daily living, such as: personal care, lifting, reading, work, driving, sleeping, recreational activities, pain intensity, concentration and headache. The questions are measured on a six-point scale from 0 (no disability) to 5 (full disability). The numeric response for each item is summed for a total score ranging from 0 to 50 [32]. A higher NDI score indicates a greater patient's perceived disability. The reliability (intraclass correlation coefficient [ICC]: 0.73 to 0.98), construct validity, and responsiveness to change have all been demonstrated in various populations [32]. For patients with cervical radiculopathy, the minimal detectable change is 10 points, and the clinically important difference is 7 points [33]. The validated French version of the NDI will be used [34].

*Disabilities of the Arm, Shoulder and Hand: *The *Quick*DASH is a region-specific questionnaire that evaluates the physical disability and symptoms of the upper limb in patients with upper extremity disorders [35]. It consists of 11 items extracted from the original 30-item DASH. Each item has five response choices, ranging from "no difficulty or no symptoms" to "unable to perform activity or very severe symptoms," and is scored on a one-to-five scale. To calculate a *Quick*DASH score at least 10 of the 11 items must be completed. The score ranges from 0 (no disability) to 100 (most severe disability). The validity, reliability (ICC = 0.96) and responsiveness of the *Quick*DASH have been established [36]. The minimal detectable change is 10.8% and the clinically important difference is 10.2% [36]. The *Quick*DASH is also valid for the measurement of upper extremity disability in patients presenting neck pain [37]. The validated French-Canadian version of the *Quick*DASH will be used [38].

*Numerical Pain Rating Scale: *The level of upper limb and neck pain will be captured separately with the NPRS. Using an 11-point scale, ranging from 0 (no pain) to 10 (worst pain imaginable), participants will be asked to answer the following question: "*On a scale of 0 to 10, where 0 corresponds to no pain and 10 to the worst imaginable pain, evaluate the intensity of your neck pain at this moment*". The same question will be asked for the upper limb. The NPRS is frequently used in clinical studies in association with the NDI [22,39,40]. The NPRS is moderately reliable (ICC = 0.76) [39], and has a clinically important difference of 20% [40].

*Cervicothoracic Mobility: *The CROM measures cervical range of motion for rotation, flexion/extension, and lateral flexion using three separate inclinometers attached to a frame similar to eyeglasses: one inclinometer in the transverse plane for rotation, one inclinometer in the sagittal plane for flexion/extension, and one in the frontal plane for lateral flexion [41]. The rotation inclinometer has a magnetic needle, whereas the flexion/extension and the lateral flexion inclinometers have gravity needles. All subjects will be seated in a standardised position during the examination. Three measures will be conducted in each direction and the means will be used for data analysis. The reliability (ICC = 0.89-0.90) and construct validity of the CROM have been established [41].

*Global Rating of Change: *GRC questions are designed to quantify a patient's perceived improvement or deterioration over time. Using a 15-point GRC scale, ranging from -7 (a very great deal worse) to 0 (about the same) to +7 (a very great deal better), participants will be asked to answer the following question: "*Overall, has there been any change in your condition since the initial evaluation? Please indicate if there has been any change in your condition by choosing one of the following options*" [42]. The validity, reliability (ICC = 0.90) and responsiveness of GRC scales have been established [43].

### Rehabilitation Programs (independent variables)

Physiotherapists working in six physiotherapy clinics in the Quebec City area will provide the rehabilitation programs. Each participant will receive eight physiotherapy treatment sessions during a 4-week span (2 sessions/week; 30 to 45 minutes each session) and will perform a home exercise program. At the beginning of each session, the physiotherapist will conduct a standardised biomechanical examination of the cervicothoracic spine. It will consist of an intervertebral segmental mobility evaluation using lateral glides at the cervical spine and postero-anterior glides at the thoracic spine (T1 to T6). Segmental mobility will be rated as normal, hypomobile or hypermobile. Sandmark & Nisell and Rey-Eiriz et al. demonstrated that this rating of cervicothoracic mobility is sensitive (> 0.80) and specific (> 0.70) [44,45]. Furthermore, this evaluation of segmental mobility is recommended in the *Neck Pain Clinical Practice Guide *of the *American Physical Therapist Association *[5]. The results of the biomechanical examination, as well as the nature and intensity of the intervention, will be documented using standardised forms.

*Conventional Rehabilitation Program: *The conventional program will consist in cervicothoracic mobilisations and stabilization exercises. This program is based on the intervention used in clinical practice [46] and on programs proposed in two RCTs evaluating individuals with neck and arm pain that do not include any specific mobilisation or exercise leading to the opening of the intervertebral foramen [22,47]. In the present study, four mobilisation techniques (10 repetitions * 30 seconds for each technique) will be executed at each treatment session. The mobilisation techniques will be chosen by the physiotherapist according to the results of the biomechanical examination performed at the beginning of each session. The physiotherapists will be allowed to use any of the following manual therapy techniques: rotations, lateral glides, postero-anterior glides, infero-medial glides or supero-anterior glides mobilisations. The physiotherapists will choose the techniques based on the patient's response to the mechanical assessment. However, the physiotherapists will not be allowed to use techniques that specifically open the intervertebral foramen of the affected segment, two segments above and two segments below. Following the mobilisation techniques, a 5-minute global manual traction (5 * 1 minute) will be applied. Although global manual traction causes an increase in diameter of the intervertebral foramen, it is not specific to the affected segment. The force for manual tractions will be the applied force causing a 50% decrease in cervical pain. Finally, a standardised home exercise program will be taught to the patients. The program, in line with best practice [48], will contain strengthening exercises for the neck flexors (longus colli, longus capitis, and rectus capitis anterior; 10 repetitions * 10 seconds, once/day in dorsal decubitus), axial extension exercises (10 repetitions * 5 seconds, once/day sitting) and one exercise chosen by the physiotherapist based on the deficits observed during the biomechanical exam. The third exercise will be chosen among: cervical or thoracic active range of motion mobility, cervical extension strengthening, or scapular strengthening. However, no exercise leading to the specific opening of the intervertebral foramen ipsilateral to the lesion will be given. Standardised postural advices will also be given.

*Rehabilitation program targeting the opening of intervertebral foramen*: The same program as for the conventional rehabilitation program will be applied, with two exceptions:

• Of the four mobilisation techniques, there will be two mandatory techniques targeting the opening of the intervertebral foramen on the same side and at the same level as the radiculopathy: global contralateral rotation mobilisation and ipsilateral lateral glide in a flexion position (10 repetitions * 30 seconds for each technique). The physiotherapist, according to the biomechanical evaluation results, will choose the two other mobilisation techniques including rotations, lateral glides, postero-anterior glides, infero-medial glides or supero-anterior glides mobilisations.

• The third exercise of the home program will be an opening technique: contralateral rotation exercise (contralateral to the affected segment; 10 repetitions * 3 seconds, 10 times/day).

Therefore, the number of mobilisation techniques and home exercises will be the same for the two intervention groups.

### Sample size and Analyses

The sample size calculation is based on the primary outcome measure, the NDI. The NDI has a clinically important difference of 7/50 (14/100) points for patients presenting a cervical radiculopathy. The standard deviation reported in the literature for this population is 18.4 [33]. The considered parameters are 0,05 for type I error (α) with a power of 0,80 (1-β). For an analysis of variance (ANOVA), the sample size required is 16 subjects per group. However, 18 subjects per group will be recruited to compensate for withdrawals and probable loss of follow-up. This sample size will provide sufficient power to detect a clinically important difference between the two groups.

Descriptive statistics (mean, standard deviation, median frequency counts) will be calculated for all outcome measures at the different times of measurement (week 0, 4 and 8) to summarise results. Baseline demographic data will be compared (independent Student t-tests and Chi-squared tests) across groups to establish the comparability of covariables. If needed, statistical adjustments will be made for baseline characteristics that are significantly different between groups. All data will be tested to ensure they meet the assumptions for the inferential statistical analyses. If they do not meet the necessary assumptions, appropriate non-parametric procedures will be used. An intention-to-treat analysis will be used in which all participants will be analysed in the group to which they were originally assigned. All dropouts and the reason for dropping out of the study will be reported. Any harms or unintended effects during the rehabilitation programs will be recorded, reported and discussed.

A mixed-model, 2-way ANOVA (Groups [experimental program, conventional intervention] × Evaluation [week 0, 4 and 8]) will be used to analyse the effects of the rehabilitation programs. Separate analyses will be conducted on each of the primary (NDI) and secondary (*Quick*DASH, NPRS, cervicothoracic range of motion) outcomes. If an interaction is detected (*P *< 0.05), simple effects will be examined. The Kolmogorov-Smirnov test (*P *< 0.05) will be conducted on the different scores to ensure normality for all variables with significant main effects. For normally distributed variables with significant main effects, post hoc dependent Student *t *tests will be conducted and effect sizes (Cohen's *d*) will be calculated. Effect sizes will be interpreted as small (0.20), medium (0.50), or large (0.80). For any variables that will not be normally distributed, the Wilcoxon signed-rank test and Glass's delta (effect size) will be used for post hoc contrasts. Since this study is looking at a new rehabilitation program, patient's perceived change following the programs will also be categorised as either success or failure. Success will be defined as a 50% improvement in NDI score and a GRC score rated as "moderately better" (+3) or higher. Patients will be classified as failure if the change on the GRC is "somewhat better" (+2) or at any level below this or if there is not a 50% improvement in the NDI [49]. The proportion of success/failure will be compared across groups (Chi-squared tests).

## Discussion

Based on the important incapacities related to cervical radiculopathy, control trials are urgently needed to define ideal intervention approaches in rehabilitation for this population. Recent systematic reviews have highlighted the lack of such trials, and thus, the need to establish the effectiveness of rehabilitation approaches. The rational for the need to determine the effectiveness of a rehabilitation program targeting the opening of intervertebral foramen of the affected segment was presented. This RCT will be the first study that directly compares a rehabilitation program targeting the opening of intervertebral foramen to a conventional rehabilitation program for patients with cervical radiculopathy. The results of this study may help to establish best clinical practice guidelines for this patient population.

## List of Abbreviations

ANOVA: analysis of variance; CIRRIS: Center for Interdisciplinary Research in Rehabilitation and Social Integration; GRC: global rating of change; ICC: intraclass correlation coefficient; LR+: positive likelihood ratio; *Quick*DASH: *Disabilities of the Arm, Shoulder and Hand *questionnaire; NDI: Neck Disability Index; NPRS: numerical pain rating scale; RCT: randomised clinical trials; ULTT: upper limb tension test.

## Competing interests

The authors declare that they have no competing interests.

## Authors' contributions

The primary authors for this protocol are PL, JSR & FD. PL, JSR & FD wrote the manuscript. PL & JSR created the protocol. FD is the statistician. PL directed the publication. JSR & FD did the methodological quality assessment. All the authors read and approved the final manuscript.

## Pre-publication history

The pre-publication history for this paper can be accessed here:

http://www.biomedcentral.com/1471-2474/13/10/prepub

## Aknowledgments

Funding was provided by the Réseau Provincial de Recherche en Adaptation-Réadaptation - Fonds de recherche en santé du Québec (REPAR/FRSQ) and by the Ordre professionnel de la physiothérapie du Québec (OPPQ). The funding agency had no role in the study design, writing the manuscript, or in the decision to submit for publication.

We would like to thank Marcel Giguère and Simon Robitaille for his help in the preparation of this study protocol.
